# Bogner, Alexander (2021): *Die Epistemisierung des Politischen: Wie die Macht des Wissens die Demokratie gefährdet*: Stuttgart: Reclam. 132 Seiten. 6,00 €

**DOI:** 10.1007/s11615-021-00372-5

**Published:** 2022-01-11

**Authors:** Jörn Knobloch

**Affiliations:** Universität Lübeck, Lübeck, Deutschland

Die Verbesserung der Demokratie ist eines der Versprechen der Wissensgesellschaft. Vor allem mit dem Wissen, welches die Wissenschaft schafft, soll die gesellschaftliche Teilhabe aller vergrößert und zugleich die Vernunft in die Politik getragen werden. Durch diesen „Wissensoptimismus“ profiliert sich die Verwissenschaftlichung demokratischen Regierens, womit das Programm der Moderne vielleicht doch noch gerettet werden kann. Indes ist die Kritik an der Macht des Wissens bereits so alt wie die Moderne, wird aber durch das Essay von *Alexander Bogner* im Hinblick auf die demokratiepolitischen Konsequenzen der Wissensfokussierung erweitert. Sein Ausgangspunkt ist die aktuelle Revolte gegen das Wissen in Form von Fake News und Verschwörungstheorien. Dabei widersteht *Bogner* dem Reflex, sie einfach zu desavouieren und stellt die wichtige Frage, ob der antiwissenschaftliche Diskurs nicht eine Folge der Wissensgesellschaft selber ist. Erst die von ihr verfolgte Epistemisierung des Politischen konstruiert Dissens exklusiv als Wissenskonflikte, die sich einer politischen Auseinandersetzung mit dem Verweis auf das bessere Wissen entziehen und damit die Revolte gegen das Wissen provozieren.

Zur Begründung wird im zweiten Kapitel zunächst die Epistemisierungsthese des Politischen entwickelt. *Bogner* zeigt, wie der Streit über die Klimapolitik, die Reaktion auf die COVID-19-Pandemie, eine Impfpflicht oder die zunehmende Kriminalität durch Wissenskonflikte, also die Konkurrenz um das bessere Wissen begründet wird (S. 18). Getragen vom Ideal des richtigen Wissens verleiten Wissenskonflikte zu der Annahme, dass Dissens einfach durch besseres Wissen ausgeräumt werden könnte. Dafür bräuchte es nur mehr Forschung, die aber nur neue Unsicherheiten produzieren und damit neue Konflikte provozieren würde (S. 26). Gleichzeitig tendieren Wissenskonflikte zur Versachlichung politischer Differenzen, was deren normativ intendierte Polarisierungen ausschließt. Tatsächlich werden die Auseinandersetzungen über normative Wertefragen aber nur vermieden und die Kontrahenten reden in ihrem Streit über das bessere Wissen beständig aneinander vorbei (S. 36). Beides fördert die Entpolitisierung gesellschaftlicher Debatten und entzieht sie einer demokratischen Bearbeitung.

Im nächsten Teil wird die Epistemisierung des Politischen demokratietheoretisch eingeordnet. Da sie in einer Phase der Erosion der Demokratie auftritt, werden zunächst drei Erklärungsansätze für deren „veritable Rezession“ (S. 37) vorgestellt, die jedoch alle die genuinen demokratischen Herausforderungen der Wissensgesellschaft vernachlässigen, mit denen sich der Autor im Anschluss beschäftigt. Im Mittelpunkt steht dabei nicht mehr die Kritik der Macht-, sondern der Wissensverhältnisse wie Jason Brennans „Epistokratie“ (S. 42) oder Helmut Willkes demokratischer Expertokratie (S. 47), deren naive Annahmen eines absoluten Wahrheitsbegriffs *Bogner* diskutiert. Er zieht deshalb den Schluss, dass in der Demokratie der absolute Wahrheitsbegriff durch einen „undogmatischen Glauben an das bessere Wissen“ (S. 55) ersetzt werden muss, um legitime Wissenslösungen zu entwickeln.

Das nächste Kapitel ändert die Perspektive und setzt sich kritisch mit Vorschlägen zur Anwendung des aus der Politik und Gesellschaft entlehnten Prinzips der Offenheit auf die Wissenschaft auseinander. *Bogner *hat für solche Ideen kein Verständnis und weist Paul Feyerabends „Politisierung der Wissenschaft“ als „epistemischen Populismus“ zurück, weil dadurch die kollektiv geteilte Wirklichkeit zerstört wird (S. 63). Aus dem gleichen Grund lehnt er Bruno Latours Idee der „Vergesellschaftung der Wissensverhältnisse“ ab (S. 68). Für *Bogner* übersehen beide Neuordnungskonzepte die stabilisierende Wirkung kollektiv geteilter Festlegungen, die Erwartungssicherheit stiften und so erst effektives wie auch produktives Handeln ermöglichen (S. 73).

Nach der Kritik an den normativen Erwartungen der Epistemisierung des Politischen befasst sich *Bogner* in den letzten beiden Kapiteln mit der demokratischen Wirklichkeit einer verwissenschaftlichten Politik. Im fünften Kapitel referiert er die jüngste Kritik an der Expertokratie als Treiber des verwissenschaftlichten Regierens (S. 75–76). Gegen die auch dort problematisierte Gefahr der allen Kontrollen enthobenen Expertinnen und Experten als „Superrepräsentanten“ (S. 79) soll die Demokratisierung der Expertise zur formalen Gleichgestellung aller Erkenntnisformen beitragen. Wo aber alle Expertise demokratisch gleich ist, hätte nach *Bogner *die Demokratie über die Wahrheit gesiegt und das Zeitalter der Post-Wahrheit eingeläutet. Gleichzeitig verliert der oder die Intellektuelle als konkurrierender Wissensakteur seine kritische Funktion, nicht nur, weil die unternehmerische Universität als Refugium ausgedient hat, sondern weil die von ihm repräsentierte Verbindung von moralischen Werten und spezifischen Wissensgründen längst zum politischen Kern der Wissensgesellschaft geworden ist.

Das sechste Kapitel diskutiert abschließend die politische Reaktion auf die Epistemisierung, die populär als Tatsachenleugner eingeordnet werden. Für *Bogner* formulieren sie die Kritik an der Wissensgesellschaft als eine „Enträtselungsoffensive von unten“ (S. 102), die konkrete, anschauliche, unmittelbare Erfahrung gegen die Abstraktion und das rationalistische Weltbild in Stellung bringen. Sie appellieren gegen die Kolonisierung der Politik durch die Wissenschaft und auf sie angemessen zu reagieren, heißt für den Autor, sich wieder für die notwendige Trennung von Politik und Wissenschaft starkzumachen (S. 109). Politische Entscheidungen folgen einer eigenen Rationalität, die durch den Rekurs auf die Wissenschaft, dies macht die Schärfe der Wissenskonflikte deutlich, eben nicht legitimer werden können.

Die Leistung des Buches liegt in der Benennung der Grenzen der Revolution des Wissens, indem es über deren demokratietheoretische Konsequenzen aufklärt. Durch die Wiederkehr der naiven, in ihrem Kern aber gefährlichen szientistischen Hoffnung, politische und damit normative Konflikte epistemisch auflösen zu können, wird die offene und kollektive Problemlösungsfähigkeit der Demokratie nachhaltig gestört. Wenn eine wissenschaftsbasierte Wahrheitspolitik zum dominierenden Modus demokratischen Regierens in der Wissensgesellschaft wird, mag das die Begründung von Politik entlasten, aber es diskreditiert jede Opposition als einen Abfall von der Wahrheit. Damit wird der weitergehende Erkenntniswert des Buches deutlich, denn es erklärt die in den aktuellen wissenschaftlichen Krisendiagnosen festgestellte Zuspitzung politischer Differenzen, die nicht mehr demokratisch zu bewältigen sind. Populismus und Illiberalismus sind demnach keine von außen über die Demokratie hereinbrechenden Phänomene, sondern Reaktionen auf die gescheiterten Versprechen der Wissensgesellschaft. *Bogners* Buch öffnet diese analytische Perspektive, gleichwohl es noch nicht erklärt, warum existenzielle Fragen demokratischer Gesellschaften heute allein im Modus szientistischer und nicht mehr politischer Überzeugungen beantwortet werden können.

